# Viscoelastic Testing for Hemostatic Assessment in Liver Transplantation

**DOI:** 10.3390/jcm15207873

**Published:** 2026-10-13

**Authors:** Asad H. Bashir, Chris Giordano, Meghan Brennan

**Affiliations:** Department of Anesthesiology, University of Florida College of Medicine, Gainesville, FL 32608, USA; cgiordano@anest.uf.edu (C.G.); mbrennan@anest.ufl.edu (M.B.)

**Keywords:** viscoelastic testing, coagulation, liver transplant

## Abstract

**Background/Objectives**: End-stage liver disease leads to a unique coagulation profile. This rebalanced coagulation status is characterized by the propensity to bleed easily and form clots in blood vessels. Managing these patients requires close monitoring and an understanding of the underlying pathophysiology. Decreased production of coagulation factors by the liver and splenic sequestration of platelets lead to conditions favoring a hypocoagulable picture. Increased levels of factor VIII and von Willebrand factor along with decreased production of anticlotting factors lead to an environment favoring thrombosis. Viscoelastic testing allows clinicians to navigate this challenging coagulation scenario and preserve the delicate balance. **Methods**: We review recent technological advancements in coagulation monitoring during liver transplantation. Viscoelastic testing has been used and validated in liver transplantation initially using mechanical methods for assessing clot formation and strength, but newer viscoelastic testing platforms are transitioning toward resonance-based technologies and cartridge-based point-of-care devices that allow for faster assessment of coagulopathies. Development of faster point-of-care devices is beneficial for liver transplantation given acute coagulopathies that can occur throughout phases of the case. **Results**/**Conclusions**: This paper will discuss briefly transfusion thresholds; how the SEER-based Quantra, resonance-based TEG 6s, and automated cartridge-based ROTEM SIGMA work; gaps using conventional coagulation testing; and the integration of these systems into perioperative decision making to support goal-directed transfusion strategies.

## 1. Introduction

Patients with acute or chronic end-stage liver disease (ESLD) presenting for a liver transplant have impairments in both procoagulant and anticoagulant pathways. Procoagulant factors, including II, V, VII, IX, X, and XI, are reduced; however, endogenous anticoagulants such as protein C, protein S, antithrombin, and tissue factor pathway inhibitor are also decreased. Evaluation of coagulation in this setting is complex and not adequately characterized by a single laboratory value [1,2,3]. Additionally, each phase of a liver transplant has distinct coagulation changes that further modify a patient’s baseline levels [1,4]. The dynamic, surgically induced changes in combination with the coagulopathies associated with liver disease are not fully characterized by conventional coagulation tests, which has led to the development and integration of whole blood viscoelastic testing (VET) in liver transplant to better understand clot formation, strength, and fibrinolysis in real time.

Accordingly, coagulation monitoring during liver transplant increasingly uses a multimodal approach integrating conventional laboratory tests with VET. Thromboelastography (TEG) (Haemon tics, Boston, MA) was first described in 1948 and has been used in liver transplantation since the 1980s [4]. Other platforms were developed, such as rotational thermoelectrometry (ROTEM) and the newer Quantra system [1,4,5]. Viscoelastic testing provides a more dynamic assessment of clot initiation, clot strength, and fibrinolysis than prothrombin time (PT)/international normalized ratio (INR) or activated partial thromboplastic time (apt) alone. Additionally, its use in liver transplantation is associated with lower blood product use and improved outcomes [1,4,6,7,8]. Previously, VET used mechanical rotational force to assess clotting properties and required blood processing in a central laboratory. This could result in significant processing time, making them less useful for intraoperative use. Newer cartridge-based point-of-care VET platforms require minimal manual preparation, allowing for intraoperative use. Additionally, these point-of-care (POC) VET systems use different technology. The Quantra system (Hemozoins LLC, Charlottesville, VA) uses sonic estimation of elasticity via resonance (SEER) to estimate clot formation and strength. TEG 6s is also cartridge based and uses resonance-based detection of viscoelastic clot properties. ROTEM sigma (Instrumentation Laboratory, Bedford, MA) also uses an automated cartridge-based system; however, it continues to use rotational thermoelectrometry in the condensed POC system.

This paper reviews recent technological advancements in coagulation monitoring during liver transplantation. Viscoelastic testing has been used and validated in liver transplantation initially using mechanical methods for assessing clot formation and strength, but newer VET platforms are transitioning toward resonance-based technologies and cartridge-based POC devices that allow for faster assessment of coagulopathies. Development of faster POC devices is beneficial in liver transplant given acute coagulopathies that can occur throughout phases of the case. This paper will briefly discuss (1) transfusion thresholds; (2) how the SEER-based Quantra, resonance-based TEG 6s, and automated cartridge-based ROTEM SIGMA work; (3) gaps using conventional coagulation testing; (4) the integration of these systems into perioperative decision making to support goal-directed transfusion strategies; and (5) limitations of these devices.

The literature reviewed for this study was chosen to provide a dedicated overview of coagulation monitoring and transfusion management during liver transplantation. References were selected based on their relevance to the use of conventional coagulation tests, VET, VET-guided transfusion algorithms, and thrombotic complications associated with coagulation management in liver transplantation. The identified literature includes clinical practice guidelines, expert guidelines, randomized and observational studies, systematic reviews, and meta-analyses. Timeline of publications spanned from 2010 to 2025, incorporating both foundational studies and recent evidence.

A restrictive, goal-directed blood product transfusion strategy has been shown to be effective for resuscitation in liver transplant and is associated with improved patient outcomes [1,4,7,8]. Blood component transfusion thresholds have different resuscitation approaches or metrics guiding their replacement, with red blood cell transfusion thresholds being the most straightforward; the current accepted transfusion threshold is for hemoglobin below 7 g/dL. Currently, none of the available POC VETs provide hemoglobin or hematocrit values; thus, providers rely on additional blood gas assessments or complete blood count lab values. Fresh frozen plasma (FFP) is a much more heterogenous transfusion practice that has been gradually adopted into a more systematic and metric-based approach using VETs. Historically, FFP has been administered within a cookbook ratio approach whereby practitioners would transfuse FFP based on the amounts of red blood cells given or expected to be given. Alternatively, the surgical field was visually evaluated for clot creation to approve for adequate coagulation enzymes and substrate. Over the last 2 decades, VET has made a resurgence in transfusion practice and technological applications. Many current practices have adopted the combination of FFP transfusion approaches by integrating the VET, cookbook ratio, and surgical field to guide plasma replacement. Viscoelastic testing has also been adopted in evaluating the remaining blood components, namely, fibrinogen and platelets, to complement the historical practice of using strictly numerical thresholds to guide transfusion: fibrinogen replacement with cryoprecipitate when plasma fibrinogen is below 100 to 150 mg/L and platelet transfusion for platelet count below 30,000 to 50,000 when active bleeding is present [1,2,9]. These numerical thresholds in isolation often failed to acknowledge the quality of the clot substrate for better or worse, as both fibrinogen and platelets have numerous qualities that can alter their facility to clot.

## 2. Conventional Coagulation Tests

When using conventional laboratory testing, multiple parameters are needed to evaluate the patient’s coagulation system, and during liver transplantation, all combinations of these still provide an incomplete profile. Prothrombin time and INR assess the in vitro extrinsic pathway (the initiation phase in the cell-based model of coagulation), reflecting the levels of factors II, V, VII, X, and fibrinogen [2,10]. Despite the fact that INR is incorporated into the Model for End-Stage Liver Disease (MELD) score because of its reflection of hepatic synthetic function, it was validated to assess anticoagulation in patients receiving warfarin [2,10]. However, INR does not account for endogenous anticoagulants produced by the liver, including protein C, protein S, and antithrombin [2,3,10]. Additionally prolonged INR in cirrhosis does not reliably predict bleeding risk, as patients with cirrhosis and elevated INR values may actually maintain normal or increased thrombin production [3,10]. The INR should be interpreted in combination with the visualization of surgical field bleeding and VET testing, if at all [1,10]. Activated partial thromboplastin time evaluates the in vitro intrinsic pathway (the amplification phase in the cell-based model of coagulation), specifically factors VIII, IX, XI, XII, X, V, II, and fibrinogen [2,10]. It is frequently prolonged in patients with ESLD and does not completely describe bleeding risk, like PT/INR [2,3,10]. During a liver transplant, apt may be useful to monitor the degree of anticoagulation if heparin is needed for extracorporeal membrane oxygenation, vascular anastomosis, or aberrant clotting. It may also indicate the release of heparin-like substances or heparin from the graft during reperfusion [1,4].

Fibrinogen decreases during liver transplantation because of reduced hepatic production, surgical blood loss, hemodilution, consumption, and hyperfibrinolysis [1,2,4]. Assessing fibrinogen concentration, fibrin-based clot strength, and fibrinolysis is necessary to determine how to improve coagulation and reduce bleeding [1,4]. There is no established target fibrinogen level during liver transplant. However, fibrinogen levels below 100 mg/dL are associated with increased bleeding, and replacement is generally considered when fibrinogen falls below 100 to 150 mg/dL, with higher levels often used during acute bleeding [2,9].

Thrombocytopenia is common in ESLD and results from reduced thrombopoietin production, splenic sequestration, bone marrow suppression, and increased platelet consumption [2,3]. Platelet count alone also does not provide a complete understanding of platelet function nor contribution to coagulation. Platelet function, even with low counts, may be partly preserved in ESLD because von Willebrand factor rises and elevated levels can compensate for a reduced platelet count [2,3]. In nonbleeding patients, platelet counts above 30,000 to 50,000 may be adequate for many procedures; however, during active surgical bleeding, higher functional platelet activity may be required because of ongoing consumption and dysfunction [2,3,9].

## 3. Viscoelastic Testing

Viscoelastic testing is widely used in liver transplants because it provides rapid, dynamic assessment of clot formation, clot strength, and fibrinolysis in whole blood. More recently, cartridge-based point-of-care platforms have been developed for VET that are particularly useful in the operating room. With these new platforms, results are available quickly, and providers can adjust therapy in real time as it pertains to blood component transfusion decisions [1].

## 4. Quantra

The Quantra system, the newest platform, uses ultrasound-based acoustic resonance to quantify clot viscoelastic properties. With SEER, ultrasound pulses induce small resonant motion in the blood sample. As the coagulation system activates, the oscillation pattern changes, which allows for the calculation of clotting properties over time [1,5]. The Quantra Stat cartridge, validated and approved by the United States Food and Drug Administration in liver transplant, provides parameters including (1) clotting time (CT), which reflects time to initial clot formation; (2) clot stiffness, which reflects overall clot strength; (3) fibrinogen contribution to clot stiffness (FCS), which isolates fibrinogen contribution after platelet inhibition; and (4) platelet contribution to clot stiffness (PCS), which estimates platelet contribution to total clot stiffness [5]. Quantra has demonstrated agreement with mechanical thromboelastography systems, such as TEG and ROTEM, for identifying hypofibrinogenemia and thrombocytopenia in liver transplantation [1,5]. Quantra, like TEG and ROTEM, also supports goal-directed transfusion strategies by showing the need for fibrinogen replacement, platelet transfusion, or enzymatic replacement rather than relying on conventional lab values or empiric blood product ratio administration. A prolonged CT suggests either coagulation enzymatic deficiency or the presence of an anticoagulant. Administration of FFP or potentially prothrombin complex concentrate could be considered depending on the setting, coagulopathic bleeding, or massive transfusion requirements from bleeding. If clot stiffness is reduced, FCS and PCS should then be assessed. Low FCS indicates the need for fibrinogen replacement with cryoprecipitate or fibrinogen concentrate. Low PCS with normal FCS indicates impaired platelet contribution and suggests platelet transfusion is indicated. When both FCS and PCS are low, replacement of both fibrinogen and platelets may be required. Increased clot stability to lysis (CSL) parameter is consistent with hyperfibrinolysis, in which case antifibrinolytic therapy may be indicated.

## 5. Thromboelastography (TEG)

The TEG 6s is a cartridge-based POC VET platform that uses resonance-based detection rather than the mechanical cup-and-pin system used in earlier generation TEG devices. As clot formation progresses within the cartridge, changes in the viscoelastic properties of the blood sample alter the detected resonance signal, allowing the system to generate a tracing that corresponds to hemostatic dynamics. The principal TEG parameters remain consistent with past generations, include R time, K time, alpha angle, maximum amplitude (MA), LY30, and LY60 [4]. It also evaluates fibrinogen-specific assessment using the citrated functional fibrinogen (CFF) assay, reported as CFF-MA, which estimates the fibrin-based contribution to clot strength after platelet contribution is inhibited.

R time reflects the time from test initiation to early clot formation and primarily represents clotting enzyme activity. If R time is long, it indicates coagulation enzyme deficiency or the presence of anticoagulants [4]. K time represents the time from initial clot formation to a clot amplitude of 20 mm and reflects the kinetics of clot formation, particularly fibrinogen contribution [4]. The alpha angle reflects the rate of clot formation and is reduced in hypofibrinogenemia and increased in hypercoagulable states [4]. Maximum amplitude reflects maximum clot strength and is largely determined by platelet function as well as the platelet–fibrin interaction [4]. LY30 and LY60 indicate the percentage of clot lysis at 30 and 60 min after MA, respectively, and elevated values indicate hyperfibrinolysis [4]. Thromboelastography-guided transfusion strategies in liver transplantation have been associated with reduced intraoperative blood product use, particularly FFP and platelet transfusion, compared with conventional coagulation testing based replacement [4,6,8]. TEG-guided transfusion during liver transplantation begins by identifying VET coagulopathies and correcting the aberrant findings as they correlate with the surgical field. Prolonged R time indicates delayed clot initiation due to coagulation enzyme deficiency or anticoagulants like heparin. In the setting of active bleeding, consider FFP transfusion, or, depending on degree of coagulopathy, bleeding, and volume status, consider prothrombin complex concentrate. Prolonged K time or a reduced alpha angle indicates impaired clot propagation, so consider fibrinogen replacement with cryoprecipitate or fibrinogen concentrate. Low MA reflects reduced clot strength, usually due to thrombocytopenia or platelet dysfunction, and supports platelet transfusion if there is active bleeding or significant derangement. Elevated LY30 or LY60 indicates hyperfibrinolysis. In the setting of ongoing bleeding after coagulopathies are corrected, consider antifibrinolytic therapy such as tranexamic acid or epsilon-aminocaproic acid.

## 6. Rotational Thromboelastometry (ROTEM)

The ROTEM sigma continues to use mechanical evaluation of clot properties; however, the pin rotates while the cup remains stable in this measuring system [4]. ROTEM uses separate assay channels with different activators to characterize specific components of coagulation [4]. EXTEM is activated by tissue factor and evaluates what is traditionally considered the extrinsic pathway and now the initiation phase of coagulation. Its parameters include CT; clot formation time; alpha angle; amplitudes at 5, 10, and 15 min (A5, A10, A15); maximum clot firmness; and lysis indices at 30 and 60 min (LI30, LI60) [4]. INTEM reflects the activity of those components that were considered the intrinsic pathway and now the amplification phase of the cell-based model of coagulation [4]. FIBTEM includes cytochalasin D to inhibit platelet contribution, thereby isolating fibrin-based clot firmness. Low FIBTEM maximum clot firmness suggests impaired fibrinogen contribution and indicates fibrinogen replacement in the setting of bleeding [1,2,4]. HEPTEM contains heparinase and can help identify heparin effect or heparin-like activity by comparison with INTEM [4]. As with TEG, ROTEM-guided transfusion algorithms in liver transplantation have been associated with reduced blood product administration, as well as less empiric transfusion when compared to conventional coagulation test utilization [1,4,7,8]. In addition, comparison of EXTEM with APTEM or changes in lysis parameters may help identify clinically significant hyperfibrinolysis [4].

Table S1 allows a comparison among Quantra, TEG 6s, and ROTEM.

## 7. Liver Transplantation Management

### 7.1. Dissection

During dissection, the phase of transplantation and preexisting coagulopathies related to ESLD are coupled with appreciating coagulation perturbations from surgical blood loss, ongoing endothelial injury, and platelet and fibrinogen dysfunction. All these contributing factors make the coagulation system very difficult to fully appreciate [1,4]. Patients typically present with abnormalities that reflect the severity of their liver disease. Those with higher MELD scores may have prolonged PT/INR, reduced fibrinogen concentration, and thrombocytopenia [1,2]. The likelihood and amount of blood loss is substantially impacted from chronic portal hypertension creating large venous collateralized blood vessels, episodes of spontaneous bacterial peritonitis resulting in adhesions and scarring, and any previous abdominal surgeries that also result in difficult surgical dissections [1]. If extracorporeal membrane oxygenation or venovenous bypass is used during this phase, anticoagulation may be used to reduce thrombotic risks of the circuit, which further complicates the appraisal of the coagulation system. The primary goal during this dissection phase of the liver transplantation from a transfusion standpoint is to minimize unnecessary transfusions by using VET-guided parameters to substantiate surgical field evaluation.

### 7.2. Anhepatic

The anhepatic phase transiently worsens preexisting coagulation abnormalities because of the complete absence of hepatic function as well as the potential for worsening hypothermia, acidosis, and hypocalcemia [1,4]. Coagulation enzyme levels, especially those with short half-lives such as factor VII (approximately 4–6 h), decline rapidly. Unique to this phase is the unregulated activity of tissue plasminogen activator release by vascular endothelial cells, which is usually rapidly cleared by the liver as well as downregulated by the tissue activator fibrinolysis inhibitor. These two enzymatic perturbations that occur during the anhepatic phase can lead to acute episodes of hyperfibrinolysis that peak right before reperfusion. Clot formation and immediate breakdown can be readily visible both in the surgical field and VET. Depending on the balance of dysfunctional procoagulant and anticoagulant factors, patients may become either more coagulopathic or more prone to thrombosis [1,3].

### 7.3. Reperfusion

During reperfusion, portal blood flow is unclamped from the splanchnic circulation and returns to the new liver graft, which may cause abrupt hemodynamic and coagulation changes as evidenced by the combined ingression of a stagnant splanchnic blood along with liver preservation solution that is acidotic, hypothermic, and hyperkalemic. Portal and systemic blood flow through the graft leads to release of fibrinolytics and heparin-like substances from the graft and splanchnic vessels [1,3,4]. These factors often produce sudden worsening of coagulopathies with impaired clot formation, reduced clot strength, and hyperfibrinolysis. Additional coagulopathies may occur because of ongoing breakdown of the endothelial glycocalyx and the release of additional heparinoids, clot breakdown/fibrinolysis, platelet dysfunction, and worsening metabolic derangements [1,3,4]. Viscoelastic testing is particularly useful during this phase because conventional coagulation studies take too long to adequately reflect these rapid changes [1,4,8]. When clinically significant bleeding is present, treatment may include antifibrinolytic therapy for selected patients, along with targeted replacement of enzymes, fibrinogen, and platelets guided by VET findings rather than empiric transfusion.

### 7.4. Neohepatic Phase

In the neohepatic phase, a well-functioning graft will begin to recover synthetic and regulatory function and coagulation abnormalities will gradually improve. If the graft is slow to return to function, the production coagulation enzymes will be delayed, and coagulopathic bleeding may ensue because of continued fibrinolysis, dilutional coagulopathy, platelet dysfunction, and hypofibrinogenemia coupled with mechanical bleeding [1,3,4].

### 7.5. Hypercoagulative Risk

Conversely, there is the potential for hypercoagulability during all phases of liver transplant that leads to an increased risk for portal vein or hepatic artery thrombus or other thrombotic complications [2,3,5]. At baseline, the production of endogenous anticoagulants such as protein C, protein S, and antithrombin III is reduced, and factor VIII and VWF are increased, leading to imbalances in coagulation. Thromboses may be more likely due to immobility, low flow states, surgical tissue injury with subsequent activation of tissue factors, inflammation, vascular clamp with venous stasis, and administration of hemostatic agents and blood products. In the neohepatic phase, ischemic injury after graft reperfusion can lead to endothelial activation, platelet activation, and increased thrombin generation that creates a prothrombotic environment. Additionally, graft synthetic function may produce procoagulant factors more rapidly than anticoagulant factors, leading to additional imbalances [1,5]. Coagulation should be assessed in a multimodal way and integrate the visualization of the surgical field, conventional laboratory values, and viscoelastic testing [1,4]. Transfusion thresholds should remain restrictive, with ongoing correction of reversible contributors such as hypothermia, acidosis, and hypocalcemia.

Institutions have drafted treatment algorithms to assist with blood product resuscitation based on the results of viscoelastic testing. While this standardized treatment protocol aids in the efficient analysis of data from a viscoelastic test, care must be taken when generalized protocols are applied broadly to specific patients, particularly those with ESLD who present with a rebalanced coagulation profile. A meta-analysis by de Oliveira Júnior et al. [11] noted that using algorithms for viscoelastic testing in patients undergoing liver transplantation was associated with reduced red cell transfusions and a higher demand for fibrinogen transfusion. Nguyen-Buckley et al. note in a large retrospective study that the implementation of ROTEM at their institution was associated with increased cryoprecipitate usage and an increased incidence of major thrombotic complications in liver transplants [12]. There is literature where specific thresholds are listed as triggers for transfusion of various blood product components. Soucy- Proulx and colleagues [13] have proposed a transfusion algorithm based on Quantra results. Due to the vast heterogeneity in this patient population, while such thresholds offer useful information, they cannot be determined to be the clear standard for use in deciding to transfuse a specific blood product. Institutional adjustments based on the patient population, but more importantly the concurrent use of clinical observation (such as state of the operative field) will lead to well informed decision making.

These devices while very useful have certain limitations. They do not replicate all in vivo coagulation conditions. For example, the Quantra cartridge runs at 37 °C and may not reflect the contribution of patient hypothermia to the coagulation profile. Similarly, it is insensitive to hypocalcemia, with calcium being an essential co-factor for clot formation. State of the art cartridge-based VET devices entail costs with initial procurement, supply of cartridges, and ongoing quality control and personnel training initiatives.

## 8. Conclusions

In liver transplantation, coagulation management is complex because patients with ESLD have abnormal procoagulant and anticoagulant pathways. Conventional coagulation tests such as PT/INR, apt, fibrinogen level, and platelet count provide useful but incomplete information. Viscoelastic testing offers a more dynamic assessment of clot initiation, clot strength, and fibrinolysis throughout the dissection, anhepatic, reperfusion, and neohepatic phases. A goal-directed transfusion strategy guided by VET testing shows specific need for fibrinogen, platelets, and coagulation enzymes, while also being shown to reduce unnecessary transfusions. Additionally, bleeding risk is only one aspect of ESLD, and patients may also be at risk for thrombotic complications, which may be better reflected in VET rather than conventional coagulation testing.

## Data Availability

No new data were created or analyzed in this study. Data sharing is not applicable to this article.

## Supplementary Materials

The following supporting information can be downloaded at: https://www.mdpi.com/article/10.3390/jcm15207873/s1, Table S1: Comparison of visco elastic testing (VET) modalities including Quantra (Q stat), TEG 6s (thromboelastography) and ROTEM (rotational thromboelastography).

## Abbreviations

The following abbreviations are used in this manuscript:

apt	activated partial thromboplastic time
CFF	citrated functional fibrinogen
CT	clotting time
ESLD	end-stage liver disease
FCS	fibrinogen contribution to clot stiffness
FFP	fresh frozen plasma
INR	international normalized ratio
MA	maximum amplitude
MELD	Model for End-Stage Liver Disease
PCS	platelet contribution to clot stiffness
POC	point-of-care
PT	prothrombin time
SEER	sonic estimation of elasticity via resonance
TEG	thromboelastography
VET	viscoelastic testing
