# Supplementary material for: Viscoelastic Testing for Hemostatic Assessment in Liver Transplantation
Source: J Clin Med. 2026 Oct 13;15(20):7873. doi: 10.3390/jcm15207873 (PMC13706071; doi:10.3390/jcm15207873)
Supplement: Supplementary file 1 [file jcm-15-07873-s001.zip › jcm-4608974-supplementary.pdf]

Table S1: Comparison of visco elastic testing (VET) modalities including Quantra (Q stat), TEG 6s (thromboelastography) and ROTEM (rotational thromboelastography).

Feature	Quantra® (QStat)	TEG® 6s	ROTEM® SIGMA
**Testing principle**	SEER sonorheometry; high-frequency ultrasound measures changes in clot elasticity/shear modulus	Resonance-based thromboelastography; detects changes in resonance frequency as clot formation alters viscoelastic properties	Automated rotational thromboelastometry; clot formation alters rotation/impedance of a pin within the sample
**Sample preparation**	Citrated whole blood; single-use cartridge; minimal hands-on preparation	Citrated whole blood; single-use microfluidic cartridge; minimal hands-on preparation	Citrated whole blood; fully automated cartridge containing lyophilized reagents; no manual pipetting
**Turnaround time**	Initial clot-time results ≈ 5 min; clot stiffness/fibrinogen/platelet results ≈ 12.5 min; fibrinolysis ≈ 25–60 min	RapidTEG/initial clot-strength information generally available in ~10 min; complete parameters depend on assay and lysis measurement	Initial parameters available within approximately 5–10 min; A10 at 10 min; complete MCF/lysis results require longer
**Major parameters**	CT, CTH, CTR; CS; FCS; PCS; CSL	R, K, α-angle, MA; CRT/MA; CFF-MA; LY30 (lysis cartridge)	CT, CFT, α-angle, A10/A20, MCF; LI30/LI60, ML
**Fibrinogen contribution**	Directly quantified as FCS (fibrinogen contribution to clot stiffness)	CFF assay suppresses platelet contribution using a platelet inhibitor; CFF-MA estimates functional fibrinogen contribution	FIBTEM suppresses platelet contribution with cytochalasin D; FIBTEM A5/A10/MCF assesses fibrin-based clot firmness
**Platelet contribution**	Directly quantified as PCS (platelet contribution to clot stiffness) = CS − FCS	Inferred from difference between total clot strength (MA) and functional fibrinogen contribution; not reported as a dedicated PCS parameter	Inferred from EXTEM − FIBTEM (often termed PLTEM); not a directly reported platelet-contribution parameter
**Fibrinolysis**	QStat CSL evaluates clot stability/lysis; results approximately 25–60 min	LY30 available with the lysis cartridge; measures percentage clot-strength reduction 30 min after MA	APTEM can confirm fibrinolysis by comparing with EXTEM; LI30/LI60 and ML quantify lysis
**Key distinguishing feature**	Directly separates fibrinogen and platelet contributions to	Provides traditional TEG parameters with rapid cartridge-based	Broadest traditional assay menu, including EXTEM, INTEM,

	clot stiffness in the same cartridge	resonance technology and functional fibrinogen testing	FIBTEM, APTEM and HEPTEM, allowing pathway-specific and fibrinolysis assessment

Abbreviations: SEER, sonic estimation of elasticity by resonance; CT, clotting time; CTH, clot time to a defined clot stiffness; CTR, clot time ratio; CS, clot stiffness; FCS, fibrinogen contribution to clot stiffness; PCS, platelet contribution to clot stiffness; CSL, clot stability to lysis; R, reaction time; K, clot kinetics; MA, maximum amplitude; CRT, clot rate; CFF-MA, functional fibrinogen maximum amplitude; LY30, percentage clot lysis 30 minutes after maximum amplitude; CFT, clot formation time; A5/A10/A20, clot amplitude at 5, 10, or 20 minutes; MCF, maximum clot firmness; LI30/LI60, lysis index at 30 or 60 minutes; ML, maximum lysis; FIBTEM, fibrin-based thromboelastometry; EXTEM, extrinsic pathway thromboelastometry; INTEM, intrinsic pathway thromboelastometry; APTEM, aprotinin-modified thromboelastometry; HEPTEM, heparinase-modified thromboelastometry; and PLTEM, platelet contribution to clot firmnes.
